# Value of passive anterior tibial subluxation on axial MRI in identifying anterior cruciate ligament functional deficiency in patients with advanced anteromedial osteoarthritis of the knee: a case-control study

**DOI:** 10.1186/s12891-021-04328-z

**Published:** 2021-05-13

**Authors:** Yuzhang Tao, Siying Tang, Pei Zhao, Wenlong Yan, Aiguo Zhou, Jian Zhang

**Affiliations:** 1 Department of Orthopaedics, The First Affiliated Hospital of Chongqing Medical University, Chongqing, 400016 China; 2 Department of Clinical Nutrition, The First Affiliated Hospital of Chongqing Medical University, Chongqing, 400016 China

**Keywords:** Unicompartmental knee arthroplasty, Anterior cruciate ligament, Anteromedial osteoarthritis, Passive anterior tibial subluxation, MRI

## Abstract

**Background:**

A functionally deficient anterior cruciate ligament (ACL) is considered one of the contraindications in unicompartmental knee arthroplasty (UKA). But there is still a lack of standardized and reproducible methods to assess ACL functional integrity in patients with advanced anteromedial osteoarthritis of the knee (AMOA). This study explores the value of passive anterior tibial subluxation (PATS) on axial MRI in evaluating ACL status in this population.

**Methods:**

Patients who met UKA indications between November 2017 and September 2020 were included and grouped into “intact” (ACLI) or “deficient” (ACLD) group according to their ACL status during surgery. All participants underwent MRI with a standardized protocol. The measurements of medial and lateral PATS were conducted on axial MRI, and the mean of them was calculated as global PATS. Then the reliability and diagnostic ability of PATS were determined.

**Results:**

A total of 85 patients (45 for ACLI group, 40 for ACLD group) were included after selection. The measurements of PATS showed excellent intra- and inter-observer reliabilities (with an intraclass correlation coefficient of at least 0.986). The global PATS of the ACLI group was significantly lower than that of the ACLD group (− 2.30 ± 1.96 vs. 1.03 ± 1.96 mm, *P*<0.0001). The diagnostic ability of global PATS was good (area under the curve = 0.897), and a threshold of 1.2 mm had a specificity of 100%, a sensitivity of 55%, and an accuracy of 78.82%.

**Conclusion:**

An axial global PATS of 1.2 mm on MRI is greatly specific for identifying a functionally deficient ACL in patients with advanced AMOA.

## Background

Anteromedial osteoarthritis of the knee (AMOA) is one of the most common degenerative changes in the elderly population [1]. Compared with total knee arthroplasty (TKA), unicompartmental knee arthroplasty (UKA) addresses the issue of advanced AMOA with better function, faster recovery, fewer complications, and retained proprioception [2]. Better clinical outcome of UKA comes with appropriate patient selection, in which patients with lateral compartment cartilage defect, functionally deficient anterior cruciate ligament (ACL), and severe knee deformity are contraindicated for Oxford UKA [3, 4]. Given that a functionally intact ACL is correlated with a longer UKA implant survival [5], a comprehensive evaluation of ACL status is crucial in determining UKA candidates.

Preoperative physical examinations (PE) for ACL integrity evaluation such as the anterior drawer test, the Lachman’s test, and the pivot shift test, are usually effective in patients with an acute ACL injury but of limited value in diagnosing chronic ACL damage in patients with degenerative knee due to the stiffness of periarticular structure [6, 7]. Direct observation of ACL’s texture and continuity on magnetic resonance imaging (MRI) in the degenerative knee may exaggerate the extent of ACL injury and exclude the potential UKA candidates with functionally intact ACL [8, 9]. In addition, inadequate quality of scanning and imaging could make ACL not optimally seen, thus lowering the diagnostic performance of MRI. Although the presence of posterior tibial bony erosion on the lateral radiograph is an indicator for identifying insufficient ACLs, this parameter is hard to quantify and subject to subjectivity and knee malposition [6, 10, 11].

In the setting of an acute complete ACL tear, the anterior subluxation of the tibia relative to the femur is prominent compared to that of intact ACL [12, 13]. The quantitative parameter for assessing the degree of such subluxation is generally called the passive anterior tibial subluxation (PATS) [14, 15]. PATS measured on the axial sequence of MRI is more reliable compared with sagittal sequence, and a threshold of 3.5 mm for axial global PATS is highly specific for diagnosing a complete ACL tear [13]. However, the ability of axial PATS to assess ACL integrity in patients with AMOA is unknown for the moment.

Considering the high sensitivity of MRI on detecting ACL injury in degenerative knees [8], it is necessary to develop a method with an acceptable specificity for a comprehensive evaluation of ACL integrity before UKA. The aim of this study was to determine (1) how axial PATS on MRI of ACL functionally intact knees compare to that of ACL-deficient knees, and (2) the threshold for PATS to evaluate ACL integrity in patients with AMOA. The hypothesis of the study is that axial PATS on MRI can be used to identify the functionally deficient ACL in patients with advanced AMOA.

## Methods

### Study population

After approval was obtained by the ethics committee of the First Affiliated Hospital of Chongqing Medical University, this retrospective case-control study was performed in a tertiary medical center. From the Electronic Medical Record System and the Radiological Database, included patients were determined based on UKA indications between November 2017 and September 2020. Then participants were grouped into “ACL functionally intact (ACLI)” or “ACL functionally deficient (ACLD)” according to their ACL status during surgery.

Inclusion criteria were (1) patients diagnosed with advanced AMOA who met the indications for UKA proposed by Hamilton et al. [4], (2) patients who had a preoperative MRI examination of the affected knee due to UKA candidates screening. Patients who met at least one of the following criteria were excluded: (1) obvious complete ACL tear on MRI, (2) ACL status during surgery not tested and recorded, (3) prominent degenerative changes in the patellofemoral or lateral compartment observed in the surgery, (4) diagnosis with spontaneous osteonecrosis of the knee (SONK). The ACL function of each patient indicated for UKA was assessed in the surgery by experienced senior surgeons. The ACL was regarded functionally intact if the ligament was present and not friable or fragmented. If the integrity of the ACL was not sure, a ligament hook was used to check whether the ACL can resist the anterior force applied by the hook [16]. The patients with adequate ACL resistance were proceeded to UKA and grouped as ACLI. Otherwise, the patients were considered ACLD and transitioned to TKA.

The flexion degree of the affected knee was determined by the medical record, and the hip-knee-ankle (HKA) angle on the full-length lower extremity radiograph was measured for evaluating the degree of knee varus deformity.

### MRI protocol and measurement

The MRI was performed within 1 week prior to surgery for all included patients. All examinations were conducted with the same 1.5 T MRI scanner (Siemens Magnetom Essenza, Germany) using a standardized institutional protocol. All subjects were placed in the supine position with a cushion under the knee, holding it in extension with neutral rotation. The knee was secured in a multichannel phased-array coil to ensure a consistent lower extremity position. The entire knee joint was included in the scanning range. The coronal and sagittal planes were scanned with the T1-weighted turbo spin-echo sequence (TSE) and the proton density (PD) TSE sequence with fat suppression (FS), and the axial plane was scanned with the PD-TSE-FS sequence. The slice thickness was set to 4 mm with a slice gap of 0.5 mm, and the field of view was 160 mm with a matrix size of 512 × 512. All scanning images were saved in Digital Imaging and Communications in Medicine (DICOM) format and analyzed with DICOM viewer Osirix Lite software (version 12.0, Pixmeo).

**Fig. 1:**
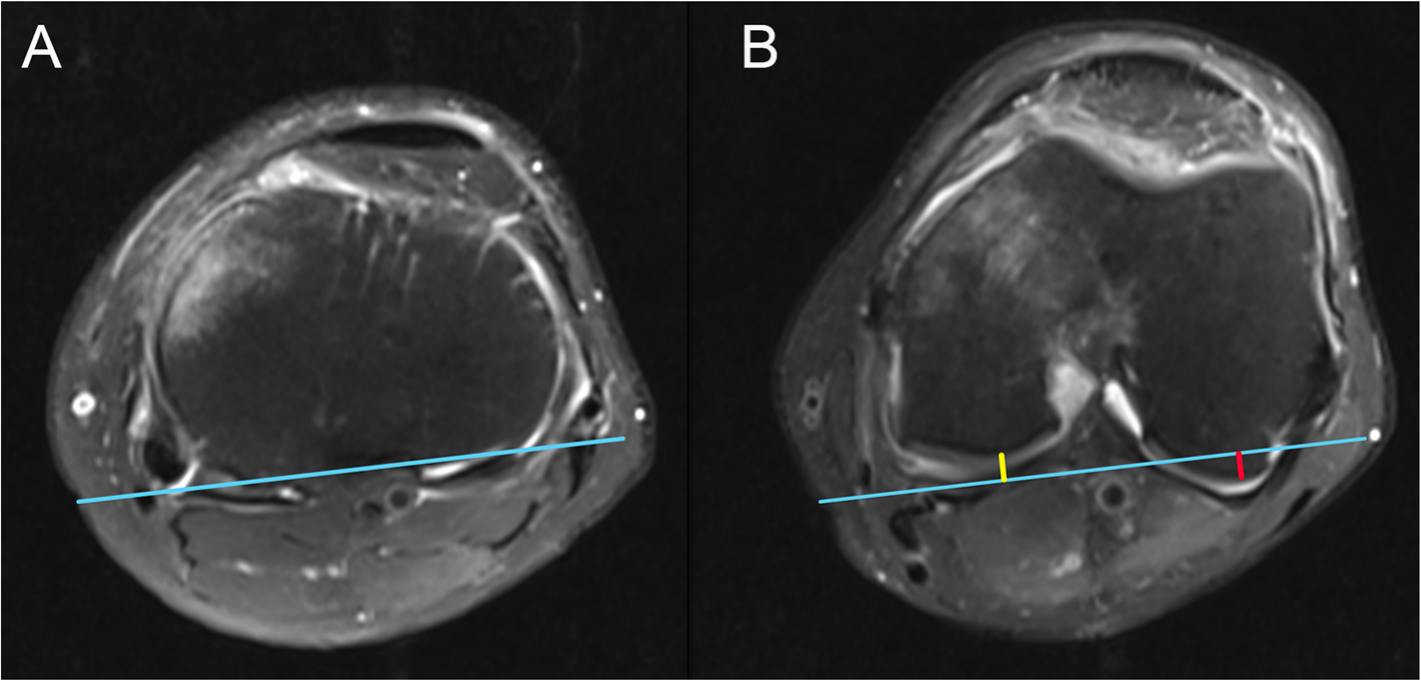
Measurements of the medial and lateral PATS. **a** Axial plane on MRI showing the line (blue) connecting the most posterior points of the tibial plateau. **b** After the most posterior points of femoral condyles are determined, the distance from both condyles to the blue line are measured. The length of the yellow line and the red line indicate the medial PATS and the lateral PATS, respectively. In this case, the value of medial PATS is negative (posterior translation), while the value of lateral PATS is positive (anterior translation). PATS, passive anterior tibial subluxation

Measurements were performed on the axial plane of MRI to evaluate the translation of the tibial plateau relative to the medial and lateral femoral condyles. According to Alexandre et al. [13], the most posterior points of the femoral condyles and the tibial plateau were determined using axial and sagittal sequences. A line was drawn through the posterior points of the tibial plateau, and the distance from the medial and the lateral condyles to this line was measured as medial PATS and lateral PATS, respectively (Fig. 1). Global PATS was calculated as the mean of the medial and lateral PATS values. The anterior translation of the tibia was expressed as a positive value of PATS, whereas the negative value of PATS indicated the posterior tibial translation.

All radiological parameters were measured independently and blindly by two orthopaedic surgeons with greater than 8 years of experience, and then the inter-observer reliability was determined. The mean of the values measured by the two observers was used for analysis. Intra-observer reliability was determined with one senior surgeon re-performing the measurements 6 weeks later.

### Statistical analysis

All statistical analyses were performed using Graphpad Prism 8.0 (Graphpad Software Inc., La Jolla, CA) and SPSS software (version 26.0; IBM, Chicago, IL). The Shapiro-Wilk test was used to determine the normality for continuous variables. The comparison of variables (expressed as mean ± standard deviation) between groups was performed using the independent t-test when the data was normally distributed. For variables with skewed distribution, the Mann-Whitney U test was used for comparison. The difference between categorical variables was assessed with the chi-square test. The reliabilities of intra- and inter-observer measurements were evaluated by the intraclass correlation coefficient (ICC), where an ICC greater than 0.75 indicated an excellent agreement. The Bland-Altman 95% limits of agreement (LOA) were also conducted for the assessment of the inter-observer agreement. The diagnostic value and optimal threshold of PATS in detecting ACL deficiency in AMOA patients were determined using the receiver operating characteristic (ROC) curve with the corresponding area under the curve (AUC). The diagnostic capacity was classified as “poor” (0.5 < AUC < 0.7), “fair” (0.7 ≤ AUC < 0.8), “good” (0.8 ≤ AUC < 0.9), and “excellent” (0.9 ≤ AUC). A *P*-value of less than 0.05 was considered statistically significant.

A post hoc power analysis using G*Power (version 3.19, Heinrich-Heine-Universitat Dusseldorf, Dusseldorf, Germany) was performed on the main parameter (global PATS) to determine the power of the study. A power of 1.00 was calculated given the effect size of 1.70, the α of 0.05, and the sample size of this study.

## Results

A total of 127 patients were screened according to the inclusion criteria. After a careful review, patients with ACL complete tear on MRI (*n* = 6), ACL status not recorded (*n* = 16), advanced lateral or patellofemoral degenerative change (*n* = 11), and SONK (*n* = 9) were excluded. Thus 85 patients were included and classified into the ACLI group (*n* = 45) or the ACLD group (*n* = 40) depending on the results of their ACL assessment in operation. The age in the ACLD group was greater than that of the counterpart, while other demographic parameters and the degree of the knee deformity showed no statistically significant difference (Table 1).

**Table 1:** Demographics and Characteristics of the Included Patients

Variables	ACLI Group (*n* = 45)	ACLD Group (*n* = 40)	*P*-value
Age (years)	64.27 ± 8.55	69.35 ± 7.90	< **0.01**
Sex (n)			0.80
Male	10	8	
Female	35	32	
Knee side (n)			0.80
Left	26	22	
Right	19	18	
Knee flexion degree (°)	112.3 ± 15.4	111.4 ± 16.0	0.79
Knee varus degree^a^ (°)	7 (5, 8)	6 (3, 7.25)	0.17

Variables with “^a^” are presented as median (interquartile range) and compared using the Mann-Whitney U test. ACLI, anterior cruciate ligament functionally intact; ACLD, anterior cruciate ligament functionally deficient. Bolded values indicate statistical significance (*P* < 0.05)

**Fig. 2:**
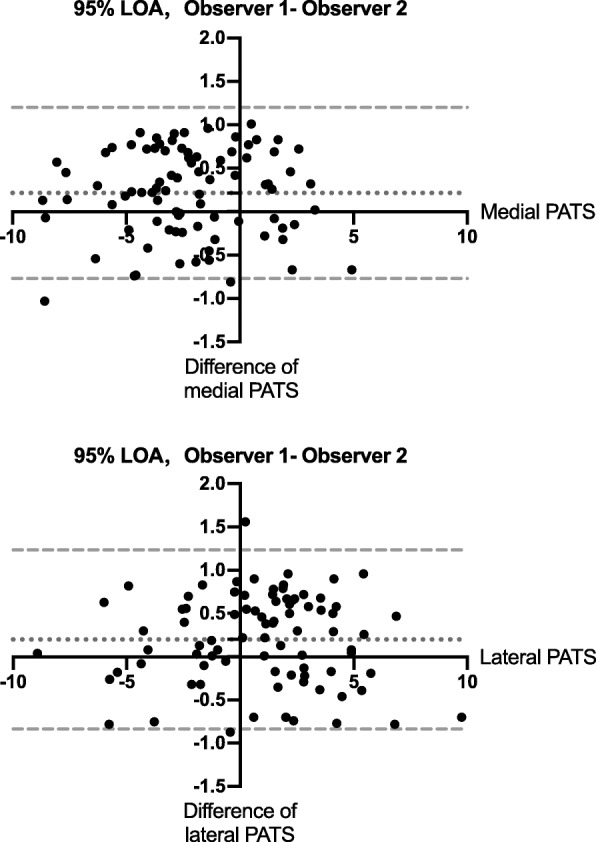
The Bland-Altman 95% limits of agreement showing the reliability of measurements between two observers. LOA, limits of agreement; PATS, passive anterior tibial subluxation

Both the measurements of medial PATS and lateral PATS showed excellent intra-observer agreements considering the ICCs were greater than 0.99 (Table 2). Inter-observer reliabilities were also satisfactory with both ICCs greater than 0.98 (Table 2) and with the Bland-Altman plot showing a difference of 0.21 ± 0.50 mm with 95% LOA from − 0.77 to 1.20 mm for the medial PATS and a difference of 0.20 mm ± 0.53 with 95% LOA from − 0.84 to 1.24 mm for the lateral PATS (Fig. 2).

**Table 2:** Intra- and Inter-observer Reliability of the Medial PATS and the Lateral PATS

	ICC	95% CI	*P* value
Intra-observer Reliability			
Medial PATS	0.993	0.990–0.996	< 0.001
Lateral PATS	0.995	0.992–0.996	< 0.001
Inter-observer Reliability			
Medial PATS	0.986	0.978–0.991	< 0.001
Lateral PATS	0.987	0.981–0.992	< 0.001

*PATS* Passive anterior tibial subluxation, *ICC* Intraclass correlation coefficient, *CI* Confidence interval

**Fig. 3:**
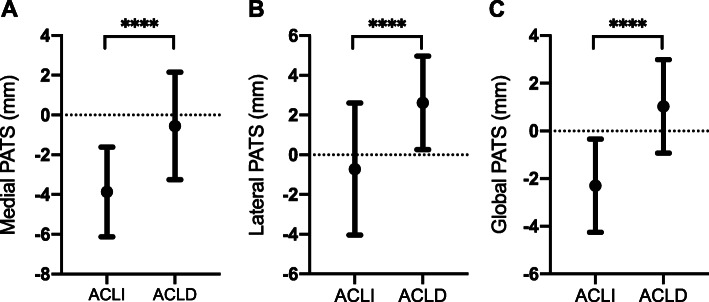
The comparison of the medial (**a**), lateral (**b**), and global (**c**) PATS between two groups (mean ± SD). PATS, passive anterior tibial subluxation; ACLI, anterior cruciate ligament functionally intact; ACLD, anterior cruciate ligament functionally deficient; SD, standard deviation. ^****^ Statistically significant difference with *P* < 0.0001

The medial (− 3.87 ± 2.26 vs. -0.55 ± 2.71 mm), lateral (− 0.72 ± 3.33 vs. 2.61 ± 2.35 mm), and global (− 2.30 ± 1.96 vs. 1.03 ± 1.96 mm) PATS of the ACLI group were all significantly lower than that of the ACLD group (Fig. 3). There was no difference in global PATS between males and females in ACLI group (− 2.03 ± 1.25 vs. -2.37 ± 2.12 mm, *P* = 0.63) and ACLD group (0.41 ± 1.67 vs. 1.08 ± 2.02 mm, *P* = 0.33).

**Fig. 4:**
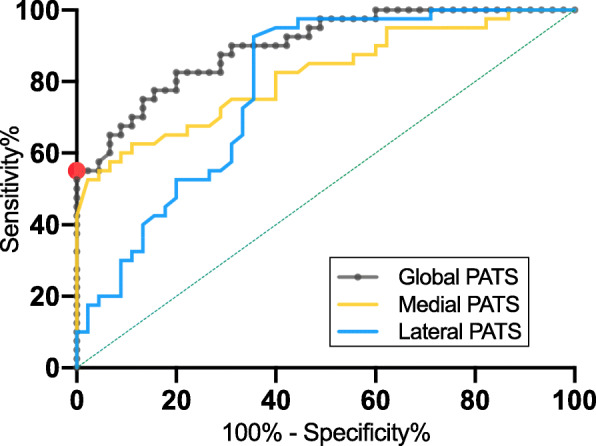
The receiver operating characteristic (ROC) curves of the medial, lateral, and global PATS. The red point indicates the threshold value of global PATS (1.2 mm) with the greatest sensitivity given a specificity of 100%. PATS, passive anterior tibial subluxation

The ROC curves of the medial, lateral, and global PATS in diagnosing ACL functional deficiency in patients with AMOA were demonstrated in Fig. 4. Three kinds of PATS showed fair or good capacities for determining ACL functional deficiency with all the AUCs greater than 0.708, while the global PATS had the greatest AUC of 0.897 (Table 3). Because the main objective of this study was to find a method with an acceptable specificity, the cut-off threshold of the global PATS was determined (1.20 mm) by finding the point with the greatest sensitivity (55%; 95% confidence interval [CI] 39.8–69.3%) while having a specificity of 100% (95% CI, 92.1–100%) on the ROC curve (Fig. 4). When the threshold of global PATS was set to 1.2 mm, the diagnostic accuracy, positive predictive value, and negative predictive value were 78.82, 100, and 71.43%, respectively.

**Table 3:** The Areas Under the ROC Curves (AUCs)

	AUC	95% CI	*P-*value
Medial PATS	0.816	0.725–0.907	< 0.0001
Lateral PATS	0.708	0.682–0.880	< 0.0001
Global PATS	0.897	0.834–0.960	< 0.0001

*ROC curve* Receiver operating characteristic curve, *PATS* Passive anterior tibial subluxation, *CI* Confidence interval

## Discussion

The current case-control study indicated that, in patients diagnosed with advanced AMOA, the medial and lateral tibial translation relative to femur on axial MRI could be reliably measured, with excellent intra- and inter-observer ICCs (> 0.98). In addition, the degree of anterior tibial translation was more prominent in the ACL functionally deficient group than the ACL functionally intact group, and the axial PATS on MRI showed good value in diagnosing ACL functional integrity for UKA candidates (with AUCs from 0.708 to 0.897). Furthermore, a threshold value of 1.2 mm for global PATS was found able to distinguish an ACL functional deficiency in this population, with a specificity of 100%, a sensitivity of 55%, and an accuracy of 78.82%.

Although an intact ACL was initially regarded as a prerequisite for UKA in 1989 [3], several pieces of literature have shown an ACL that can provide functional stability of the knee (not necessarily “macroscopically” intact) is enough for a favorable implant survival [16–18]. When a functionally deficient ACL is suspicious based on preoperative PE or MRI, orthopedic surgeons usually have to wait until surgery to make the final diagnosis, deciding whether UKA is suitable for the candidate. Therefore, TKA instruments are often on standby in case of insufficient ACL, thus leading to resource inefficiency. A reliable assessment of ACL’s status before UKA is needed to address this issue. However, there is still a lack of standardized or reproducible methods to evaluate ACL functional integrity before UKA. PATS on MRI has been moderately investigated for distinguishing a complete ACL tear in young patients suffered from acute knee injuries and showing a good diagnostic value in this population [13–15]. But in elderly patients diagnosed with advanced AMOA, the PATS, as a quantitative parameter in assessing ACL status, is little studied. This study demonstrated that an axial global PATS on MRI greater than 1.2 mm could be used to exclude a patient from UKA candidates because of a functionally deficient ACL.

Despite the fact that the anteroposterior position of bone erosion on the tibial plateau in the lateral radiograph has been shown to correlate with ACL deficiency [10] for UKA candidates, its sensitivity (36%) and specificity (79%) are relatively low [6]. Besides, the quality of lateral radiograph is subject to knee rotation, making this qualitative and subjective evaluation less reliable. The cartilage wear pattern on sagittal MRI has also been shown not useful in the diagnosis of ACL integrity in the arthritic knee [19]. In comparison, the distance measurement such as PATS on MRI is reproducible (with excellent ICCs and small inter-observer difference on Bland-Altman plot) and quantitative due to its standardized protocol, leading to a consistent assessment of the tibial translation.

The direct assessment of ACL’s texture and continuity on MRI appears to be too sensitive in detecting ligament degeneration for patients with AMOA, as Sharpe et al. [8] have described that only 13% of patients turn out to have ACL degeneration on surgical inspection whereas 33% of them show obvious ACL signal change on MRI, suggesting the potential UKA candidates may be abandoned prematurely based on direct MRI observation of ACL. They speculated that the high sensitivity might be explained by the capacity of MRI to see inside the ligament, whereas only the outer surface of ACL can be observed at the surgery. Moreover, Hurst et al. [9] have shown that abnormal preoperative MRI findings of ACL do not influence the outcome of UKA when surgical criteria are met, implying the limited value of MRI observation alone in evaluating ACL status for degenerative knees. However, an axial global PATS threshold of 1.2 mm found in this study could be used as an indirect parameter, with excellent specificity, to diagnose an ACL deficiency preoperatively, combined with the highly sensitive MRI observation of ACL.

PATS measured on the sagittal sequence of MRI may not be consistent because of the variation in determining the line perpendicular to the tibial plateau among different observers [13]. In addition, the oblique sagittal sequence instead of the true sagittal sequence is usually implemented for a better view of ACL in routine knee MRI examination. Thus, it is more practical and reliable to assess PATS on the axial sequence of MRI. Hardy et al. [13] have assessed the ability of axial global PATS on MRI in detecting complete ACL tears in young adults (mean age 27.1 ± 1.7 years), finding a threshold of 3.5 mm with a sensitivity of 55.2%, a specificity of 100% and, an accuracy of 77.6%. Our results showed a comparable diagnostic capability with a threshold (1.2 mm) smaller than 3.5 mm but larger than 0 mm, which is reasonable since the objective of this study is to find a threshold for detecting ACL functional deficiency but not complete tears. We speculate that the moderate sensitivity (55%) of axial PATS threshold 1.2 mm found in this study might be correlated with the stiffness of peri-articular structure in AMOA patients. PATS≥1.2 mm may indicate that the extent of the patient’s ACL deficiency is prominent, then the stiffness of peri-articular structure cannot compensate the anterior translation of the tibia, leading to the high specificity of axial PATS threshold 1.2 mm. For patients with a less severe extent of ACL deficiency, the stiffness of peri-articular structure could partly compensate the tibia’s anterior translation, causing the value of their PATS to be similar to that of the ACLI population. Therefore, future studies that prospectively record patients’ knee scores are needed to explore how the stiffness of peri-articular structure would affect axial PATS in AMOA patients.

In ACL reconstruction, failure to restore an anatomical femorotibial relationship might result in suboptimal outcomes due to disturbed biomechanics [15]. Similarly, the femorotibial mismatch (PATS) may predispose patients undergoing UKA to early implant failure. However, the femorotibial translation could be altered by the variation in the slope of the tibial component in UKA [20], as Hernigou et al. [21] have found that a posterior slope of less than 5° is associated with a longer implant survival of UKA in ACL-deficient knee when compared with a posterior slope of greater than 8°. For this reason, the posterior slope of the tibial implant could be reduced appropriately to compensate for the anterior tibial subluxation caused by ACL deficiency in patients with axial PATS slightly greater than 1.2 mm. However, it is worth noting that the 1.2 mm threshold of axial PATS could be only used to exclude patients who are contraindicated for UKA because of their functionally deficient ACLs but not to decide whether the patient is suitable for UKA. More pre-operative metrics need to be combined in the future, with different weights placed for each metric, to build a new diagnostic model with a better sensitivity while maintaining 100% specificity.

To our knowledge, this is the first study to compare PATS on axial MRI between AMOA patients with different ACL statuses. However, there are several limitations to our study. First, the assessment of the ACL integrity during surgery was conducted by more than one senior surgeon, although each one followed the same protocol, which could introduce recruitment bias. Second, the age in the ACLD group was greater than the ACLI group, so that a confounding factor might have been brought in this case-control study. However, the age difference could be explained by the strong association between the grade of ACL degeneration and the grade of knee osteoarthritis [22], where a greater age is related to a greater degeneration of the knee [23, 24]. Third, the slice thickness of the MRI was 4 mm, which could create bias in PATS measurements.

## Conclusions

This study provides a quantitative method to assess ACL status preoperatively in UKA candidates. An axial global PATS of 1.2 mm on MRI was shown able to identify a functionally deficient ACL with excellent specificity, which is helpful to exclude a patient from UKA candidate.

## Data Availability

The datasets used in this study are available from the corresponding author upon reasonable request.

## Abbreviations

ACL: Anterior cruciate ligament
UKA: Unicompartmental knee arthroplasty
AMOA: Anteromedial osteoarthritis of the knee
PATS: Passive anterior tibial subluxation
ACLI: ACL functionally intact
ACLD: ACL functionally deficient
MRI: Magnetic resonance imaging
TKA: Total knee arthroplasty
SONK: Spontaneous osteonecrosis of the knee
HKA: Hip-knee-ankle
TSE: Turbo spin-echo sequence
PD: Proton density
FS: Fat suppression
DICOM: Digital Imaging and Communications in Medicine
ICC: Intraclass correlation coefficient
LOA: Limits of agreement
ROC: Receiver operating characteristic
AUC: Area under the curve
